# Headache and *NOTCH3* Gene Variants in Patients with CADASIL

**DOI:** 10.3390/neurolint15040078

**Published:** 2023-10-09

**Authors:** Oliwia Szymanowicz, Izabela Korczowska-Łącka, Bartosz Słowikowski, Małgorzata Wiszniewska, Ada Piotrowska, Ulyana Goutor, Paweł P. Jagodziński, Wojciech Kozubski, Jolanta Dorszewska

**Affiliations:** 1 Laboratory of Neurobiology, Department of Neurology, Poznan University of Medical Sciences, 61-701 Poznan, Poland; 76624@student.ump.edu.pl (O.S.); ikorcz@post.pl (I.K.-Ł.); 86648@student.ump.edu.pl (U.G.); 2 Department of Biochemistry and Molecular Biology, Poznan University of Medical Sciences, 61-701 Poznan, Poland; bslowikowski@ump.edu.pl (B.S.); pjagodzi@ump.edu.pl (P.P.J.); 3 Faculty of Health Care, Stanislaw Staszic University of Applied Sciences in Pila, 64-920 Pila, Poland; mpwisz@gmail.com; 4 Department of Neurology, Specialistic Hospital in Pila, 64-920 Pila, Poland; 5 Chair and Department of Neurology, Poznan University of Medical Sciences, 61-701 Poznan, Poland; piotrowska.ada@ump.edu.pl (A.P.); wkozubski@ump.edu.pl (W.K.)

**Keywords:** headache, variants of *NOTCH3* gene, CADASIL

## Abstract

Autosomal dominant cerebral arteriopathy with subcortical infarcts and leukoencephalopathy (CADASIL) is an inherited vascular disease characterized by recurrent strokes, cognitive impairment, psychiatric symptoms, apathy, and migraine. Approximately 40% of patients with CADASIL experience migraine with aura (MA). In addition to MA, CADASIL patients are described in the literature as having migraine without aura (MO) and other types of headaches. Mutations in the *NOTCH3* gene cause CADASIL. This study investigated *NOTCH3* genetic variants in CADASIL patients and their potential association with headache types. Genetic tests were performed on 30 patients with CADASIL (20 women aged 43.6 ± 11.5 and 10 men aged 39.6 ± 15.8). PCR-HRM and sequencing methods were used in the genetic study. We described three variants as pathogenic/likely pathogenic (p.Tyr189Cys, p.Arg153Cys, p.Cys144Arg) and two benign variants (p.Ala202=, p.Thr101=) in the *NOTCH3* gene and also presented the *NOTCH3* gene variant (chr19:15192257 T>G). Clinical features including headache associated with *NOTCH3* (chr19:15192257 T>G) are described for the first time. Patients with pathogenic/likely pathogenic variants had similar headache courses. People with benign variants showed a more diverse clinical picture. It seems that different *NOTCH3* variants may contribute to the differential presentation of a CADASIL headache, highlighting the diagnostic and prognostic value of headache characteristics in this disease.

## 1. Introduction

Cerebral autosomal dominant arteriopathy with subcortical infarcts and leukoencephalopathy (CADASIL) is one of the most common hereditary, progressive diseases of small cerebral vessels. CADASIL has an estimated prevalence of two and five cases per 100,000 individuals [1]. CADASIL is characterized by common clinical manifestations, including migraine with aura (MA), young to mid-age recurrent onset of ischemic stroke, psychiatric symptoms, apathy, and cognitive disorder progressing to dementia. In addition, patients with CADASIL have an increased risk of ischemic stroke and cognitive dysfunction [2,3]. A study has reported that 60–85% of CADASIL patients experience their first and subsequent recurrent ischemic stroke with lacunar syndromes between 45 and 50 years old [4]. MA, commonly an atypical aura, affects 20–40% of patients and is the first symptom of the disease, which begins in the second or third decade of life [4,5]. Moreover, MA can occur decades before a stroke. It has also been proven that a small number of CADASIL patients experience other types of headaches [6,7,8,9].

A headache refers to pain in any region of the head. Headaches may occur on one or both sides of the head, may be isolated to a certain location, radiate across the head from a distinct point, or have a viselike quality. A headache can be experienced as a sharp pain, a throbbing sensation, or a dull ache. Headaches can develop gradually or suddenly and can last from anywhere between less than an hour and several days [10,11,12]. According to the 2018 International Classification of Headache Disorders Third Edition (ICHD-3) [13], more than 200 types of headaches have been identified. Headaches can be divided into three main categories: primary headaches, secondary headaches and neuralgia. Primary headaches are those that are not caused by another disease, i.e., the headache itself is the disease. Primary headaches cause episodic and chronic head pain without an underlying pathologic process, disease, or traumatic injury. The most common are migraine and tension-type headaches (TTH) [14,15]. Secondary headaches are symptoms of other diseases (for example, brain infections such as encephalitis or brain tumors) [16].

As we highlighted above, a few CADASIL patients experience other types of headache, e.g., tension-type headache (TTH) [9,17]. A TTH is generally characterized by patients having mild to moderate pain, often described as feeling like a tight band around the head. Symptoms of a TTH include dull, aching head pain, tightness or pressure across the forehead or on the sides and back of the head, and tenderness in the scalp, neck, and shoulder muscles [18].

However, most CADASIL patients experience headache pain from a migraine. As described above, migraines belong to the primary headache category and can occur even before the onset of the first stroke in CADASIL patients [6]. Migraine headaches can last from a few minutes to many hours or days and may occur daily. According to ICHD-3, the disease has two main clinical subtypes: migraine without aura (MO), commonly known as migraine, and migraine with aura (MA) [13]. MO is the more frequent form of migraine, affecting around 70% of migraineurs [19]. Symptoms include headache pain that occurs without warning and is usually felt on one side of the head, along with nausea, confusion, mood changes, fatigue, and increased sensitivity to light or sound and noise. The second clinical subtype is migraine with aura (MA), affecting about 30% of patients with CADASIL. Patients may temporarily lose part or all of their vision. Classic symptoms include speaking trouble, an abnormal sensation, numbness, muscle weakness on one side of the body, a tingling sensation in the hands or face, and confusion [13,20,21,22,23]. The aura may occur without headache pain, which can strike anytime [13]. Also, the nature of migraine headaches can change over a patient’s lifetime (for example, during menopause) [24,25].

Despite the high incidence of migraines, its pathogenesis is still unknown. As with other neurological diseases, attention is now being paid to the roles of genes that may be involved in migraine pain [26]. The first genetic migraine studies focused on a rare monogeneous subtype of MA, namely familial hemiplegic migraine (FHM). In the pathogenesis of FHM type 1, 2, and 3, mutations in *CACNA1A*, *ATP1A2*, and *SCN1A* genes were identified, respectively [27,28,29].

As pointed out by de Vries in 2014, insights into the genetic basis of migraines may also come from other monogenic syndromes, such as CADASIL, which is caused by mutations in the *NOTCH3* gene [30]. In one particular study, patients with a mutation in the *NOTCH3* gene showed an increase in white matter hyperintensities on brain MRI, compared to controls, and MA was more common in these subjects than in their controls [31].

An analysis of several large CADASIL families has demonstrated a genetic linkage to a single disease locus on chromosome 19q12 [32]. This disease locus points to a monogenic autosomal-dominant disorder caused by a mutation in the *NOTCH3* gene, localized to chromosome 19, and consisting of 33 exons encoding the 2321 amino acid transmembrane receptor NOTCH3 [33]. The Notch family was discovered one century ago [34]. We know that mammals have four Notch receptors (Notch1, Notch2, Notch3, and Notch4) [35]. The Notch pathway leads to the expression of its target genes via translocation, post-translational modifications, and activation by its ligands. The Notch pathway leads to the expression of its target genes via translocation, post-translational modifications, and activation by its ligands. The Notch receptor, after translation, is modified in the Golgi via proteolytic cleavage at site 1 (S1) by Furin-like convertase; then, it is transported to the cell surface as a heterodimer held together by noncovalent interactions. The receptor on the signal-receiving cell is activated by ligand binding on the cell surface of a neighboring signal-sending cell. Ultimately, this induces a conformational change in the receptor to expose site 2 (S2) for cleavage by a disintegrin and metalloprotease [36,37]. Initially described in proliferating neuroepithelium, the *NOTCH3* gene and over 70% of mutations in CADASIL have been reported to occur in EGF-like repeats 3 and 4 of the Notch3 extracellular domain [38]. These are commonly missense mutations involving a cysteine residue, in which cysteine is replaced with another amino acid, but small in-frame deletions and splice-site mutations can also be found [39,40,41]. These mutations may involve some mechanisms associated with the mutant Notch3. The first mechanism is the local accumulation of the Notch3 extracellular domain in vascular smooth muscle due to local aggregation and impaired endocytosis. This prevents protein ubiquitination and degradation and leads to the formation of granular osmiophilic material [42,43]. The second mechanism involves reducing the transport of mutant Notch3 to the cell surface, subsequently resulting in intracellular aggregation. Another possibility includes mutations in EGF repeats 10 and 11 that may alter CBF1/RBP-jκ interaction. Mutant Notch3 receptors show no change in affinity for ligand binding, and some mutations may increase signaling [44]. The most common mutations in the *NOTCH3* gene and their protein products are presented in Table 1.

Genetic testing is the most sensitive and specific method for diagnosing CADASIL. Furthermore, it can provide a diagnosis at an early stage of life in asymptomatic persons and does not require an MRI [45]. Still, it is difficult to determine the patient’s clinical course because they can progress differently, even if they have the same mutation, belong to the same family, or are twins [46].

This study aimed to observe the presence or absence of a correlation between selected genetic variants of the *NOTCH3* gene and the clinical characteristics of patients with CADASIL. This can help determine whether there are differences in the clinical picture between people with pathogenic/likely pathogenic changes and people with benign changes in the *NOTCH3* gene. For this purpose, exons 3, 4, 5, 11, and 12 of the *NOTCH3* gene were subjected to genetic testing. The typical *NOTCH3* gene mutations are localized to exons 2–24 encoding EGF-like domains. Since most mutations are present in exon 4 of *NOTCH3*, genetic analysis should begin with screening this exon and then expand to include the rest [47]. Here, we report pathogenic, likely pathogenic, and benign changes in the selected exons of the *NOTCH3* gene in patients with CADASIL and suspected or diagnosed CADASIL syndrome.

## 2. Patients and Methods

### 2.1. Patients

Throughout the period from 2017 to 2023, 53 people (34 women, 19 men) with suspected CADASIL were selected. Participants were hospitalized in the Clinical Hospital of Heliodor Święcicki in Poznań and the Specialist Hospital Stanisław Staszic in Piła. The neurobiologist referred these participants to the Neurobiology Laboratory Department of Neurology at Poznan University of Medical Sciences (PUMS) for genetic testing to detect genetic variants in the *NOTCH3* gene. Genetic changes in this gene were confirmed in 30 subjects (20 females, 10 males; 43.6 ± 11.5, 39.6 ± 15.8, respectively). Of the 30 subjects, 14 were diagnosed with MA, 3 were diagnosed with MO, 12 were diagnosed with another type of headache (e.g., TTH or other headache), and 1 subject was asymptomatic, according to the 2018 International Classification of Headache Disorders Third Edition (ICHD-3) [13]. The patients underwent neuroimaging studies. Table 2 shows the demographic data regarding the study participants with a confirmed variant in the *NOTCH3* gene. This study was approved by the PUMS Local Bioethics Committee (No. 931/17 of 4 December 2017, with extension No. 971/22 of 8 December 2022, valid until 2025).

### 2.2. Genetic Analysis

The material for genetic testing was venous blood collected for the presence of disodium edetate (EDTA). The blood samples were stored at −80 °C.

The first step involved isolating genomic DNA from venous blood using the Blood Mini Plus column isolation kit (A&A Biotechnology, Gdansk, Poland). The genetic variants of the *NOTCH3* gene were analyzed via high-resolution melt analysis (HRMA) using the CFX Connect™ Real-Time system (Bio-Rad, Hercules, CA, USA). Primers for HRMA were designed using databases available on the Internet based on the published genome sequence of the *NOTCH3* gene. Variants in exons 3, 4, 5, 11, and 12 of this gene were analyzed. The primer sequences are presented in Table 3.

To improve the conditions of the HRMA process, temperature gradient PCR (MJ Mini™ Gradient Thermal Cycler, Bio-Rad, Hercules, CA, USA) was initially performed for selected pairs of primers, optimizing annealing to the DNA template. The temperature gradient involved PCR at 55 °C–65 °C, followed by 2% agarose gel electrophoresis.

Genomic DNA was used as an intercalating dye for real-time PCR with EvaGreen (SsoFast™ EvaGreen^®^ Supermix, Bio-Rad, USA). Melting analysis was performed, and the data were analyzed using Melting Analysis software (Bio-Rad, Hercules, CA, USA). DNA extraction from agarose using Gel-Out Kit (A&A Biotechnology, Gdansk, Poland) was performed to obtain the best quality material for sequencing. HRMA results were confirmed at an independent facility via Sanger sequencing using a 3130xl genetic analyzer (Applied Biosystems HITACHI, Beverly, MA, USA). The sequence reads were analyzed using the FinchTV application (Geospiza, Inc., Seattle, WA, USA) and confirmed using the ClinVar (NIH, Bethesda, MD, USA), Ensembl.org (The Ensembl Genomes Project, UK), and Varsome (Saphetor SA, Lausanne, Switzerland) databases.

### 2.3. Statistical Analyses

GraphPad (Instant) was used to evaluate the genetic results of the study, which are presented in percentage relationships. Fisher’s exact test was also used.

## 3. Results

**Figure 1:**
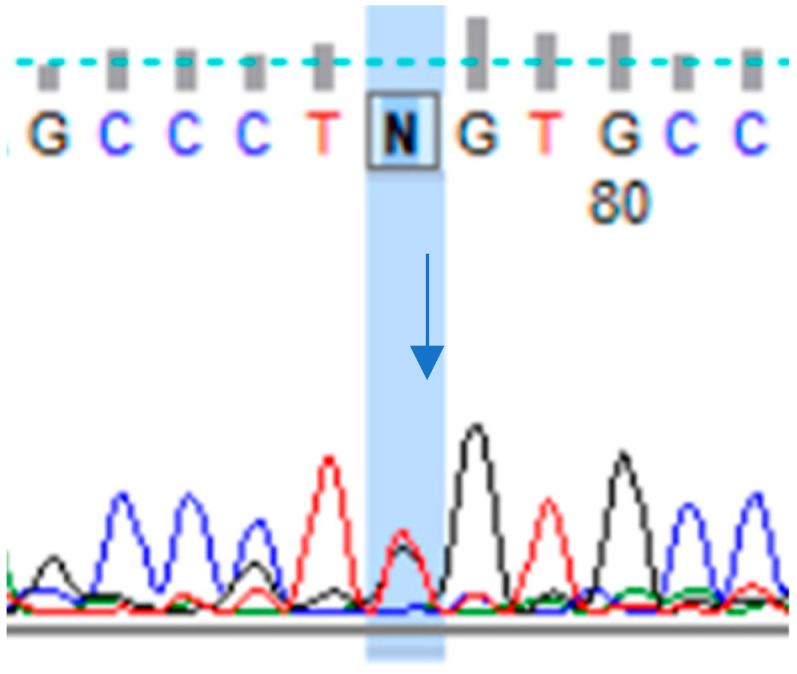
Heterozygous variant in the *NOTCH3* gene, chr19:15192257 T>G. A clinical characteristics of this variant not yet been described in the literature (analyzed according to the following database: https://varsome.com/) (accessed on 13 December 2023).

In the presence of 30 individuals, our studies showed six different *NOTCH3* genetic variants, with three being described as pathogenic/likely pathogenic (p.Tyr189Cys, p.Arg153Cys, p.Cys144Arg) and two being described as benign variants (p.Ala202=, p.Thr101=). These variants have been previously described in the literature but were examined for the first time in the Polish population in our study. Moreover, we identified the *NOTCH3* gene mutation (chr19:15192257/c.382T>G p.Cys128Gly), displayed in Figure 1, which has only been mentioned once as a “de novo mutation” in CADASIL patients in the literature to date, but this is the first time we have described the clinical features including headache associated with this variant.

According to the Varsome database (accessed on 13 December 2023), the variant chr19:15192257 T>G of the *NOTCH3* gene, identified in exon 4, is located in codon 128 and encodes a cysteine [48].

The chr19:15192257 T>G variant was found in one patient (female, 45 years old) who reported episodes of MO from the age of 34 (>10 years of MO). The MO attacks lasted for at least a few hours and occurred 1–2 times a month. In addition, numerous cases of migraines and strokes have been reported among the family members of this patient. The patient was reported to the neurological ward with symptoms of paresis of the upper limbs and increasing pain in the left eye socket, with an earlier diagnosis of strabismus. Imaging studies (MRI, CT) showed no changes in vascular origin.

The pathogenic variant p.Tyr189Cys (c.566A>G) of the *NOTCH3* gene was identified in exon 4, representing a classic pathogenic CADASIL mutation involving a change in the number of cysteine residues from six to seven [49]. This variant was detected in three women belonging to one family. All three subjects (100%) had a common clinical characterization: MA, the onset of migraine around 20 years of age, migraines triggered following highly stressful situations (severe stress in life, partum, pregnancy), attacks lasting approximately two days, 1–2 times or 3–4 times per month with changes of vascular origin in the MRI/CT image.

In one person, out of 30 subjects, the presence of the p.Arg153Cys variant (c.457C>T, rs797045014) of the *NOTCH3* gene was found. In this variant, the A allele was observed instead of the standard G allele, resulting in the addition of an arginine instead of a cysteine in the protein’s amino acid sequence [50]. This variant was identified in a 63-year-old woman with headaches lasting less than 24 h and occurring 1–2 times a month. The CT scan showed numerous ischemic changes in the periventricular white matter. In this patient, along with the pathogenic variant, the benign p.Thr101= variant was also detected.

In exon 4, a likely pathogenic variant, p.Cys144Arg (c.1630C>T, rs2046934287), was also identified. According to the ClinVar database, this variant breaks a cysteine residue in the EGF-like repeat domain, which is important for the structure of the protein [51]. The p.Cys144Arg variant was identified in a 53-year-old female. This patient was diagnosed with headaches >4 times per month. In addition, neuroimaging studies showed extensive hypodense changes in the brain’s white matter. The patient’s medical history contained information regarding numerous strokes occurring in her family members. Two benign variants, p.Ala202= and p.Thr101=, were also detected in this patient.

The first benign variant of the *NOTCH3* gene, p.Ala202= (c.606A>G, rs1043994), was identified in sixteen patients. According to the ClinVar database (NIH, USA), this variant is a synonymous variant in exon 4 that ensures that the replacement of a single nucleotide does not result in a change in the amino acid encoded by the nucleotide sequence [52]. Of the sixteen patients with this variant, nine (57%) had MA, one had MO (6%), one had TTH (6%), four had other headaches (25%), and one was asymptomatic (6%). The headaches lasted for <24 h, 24–48 h, or >48 h in fourteen (88%), one (6%), and one (6%) subjects, respectively. Ten people (63%) had headaches 1–2 times a month, two people (12%) had headaches 3–4 times a month, and four people (25%) had headaches >4 times a month. Only three subjects (19%) reported vascular changes in neuroimaging studies (MRI/CT). Thirteen people (81%) showed no changes regarding vascular origin.

**Figure 2:**
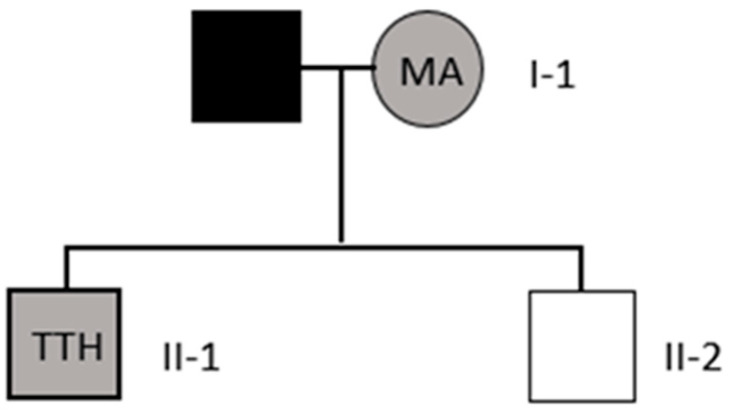
The pedigree of the Polish family with the p.Ala202= *NOTCH3* variant. Black—no data; grey—symptomatic patients; white—patients without headache.

The benign variant p.Ala202= was detected in a family of three whose members presented a different clinical picture. The mother (I-1, 46 years old) has been suffering from MA for ten years (onset at the age of 36), and her 18-year-old son (II-1) has suffered from tension-related disorders for ten years (onset at the age of 8). Both the mother and son had similar headaches, which occurred two to three times a month and lasted several minutes. However, numerous bilateral periventricular ischemic changes were found only in the mother’s computed tomography. The second son (II-2, 21 years old), who did not have headaches, was also examined (Figure 2).

The second benign variant of the *NOTCH3* gene, p.Thr101= (c.303C>T, rs3815188), was detected in exon 3. p.Thr101= is a synonymous variant, where the replacement of a single nucleotide does not result in a change in the amino acid [53]. This variant was present in eight individuals with the benign variant p.Ala202=. Of the eight patients, five (61%) had MA, one had MO (13%), one had TTH (13%), and one had other type of headache (13%). Headaches lasted for <24 h, 24–48 h, or >48 h in four subjects (50%), three subjects (37%), and one subject (13%), respectively. Three people (37%) had a headache 1–2 times a month, one person (13%) had a headache 3–4 times a month, and four people (50%) had a headache >4 times a month. Only two (25%) showed vascular changes in the neuroimaging studies (MRI/CT).

The results suggest that the pathogenic/likely pathogenic variants (p.Tyr189Cys, p.Arg153Cys, p.Cys144Arg) of *NOTCH3* were more correlated with MA, while the benign variants (p.Ala202=, p.Thr101= and p.Ala202=) appeared to be more associated with different types of headaches, including MO, tension-type headaches, and other types of headaches (Fisher’s exact test, *p* = 0.0233).

Table 4 shows the identified genetic variants of the *NOTCH3* gene and the clinical characteristics of the CADASIL patients in whom these changes were detected.

## 4. Discussion

In this study, we aimed to assess the clinical features of headaches in CADASIL patients whose marked genetic variant determined pathogenicity. Our research contributes to investigating whether MA is the first symptom of CADASIL, appearing several years before the onset of ischemic events. Singhal et al. [54] in 2004 and Narayan et al. in 2012 [55], during their CADASIL studies, focused on MA symptoms and found no relationship between the mutation site and clinical phenotype.

Three pathogenic/likely pathogenic variants, two benign variants, and one variant of the *NOTCH3* gene (chr19:15192257 T>G) have been reported in the literature [48], but the detailed clinical significance last variant and clinical picture of a patient with this variant has not been presented.

Among the three people with the pathogenic p.Tyr189Cys variant of the *NOTCH3* gene, we observed a common phenotype: MA (100%), onset at a young age (around 20 years old), onset of headaches after severe experiences (high stress, pregnancy, childbirth), attacks lasting about 2 days, 1–2 times or 3–4 times a month with changes of vascular origin in the MRI/CT image. A detailed clinical picture of this family was presented by Dorszewska et al. [49]. However, it should be noted that knowledge about the role of the pathogenic p.Tyr189Cys variant of the *NOTCH3* gene in MA and CADASIL remains limited, and further research on this variant is needed.

The second pathogenic variant reported in our study, p.Arg153Cys, is already known, primarily in the context of CADASIL [1,56,57,58,59,60]. In 2016, He et al. [57] confirmed phenotypic changes in a patient with the p.Arg153Cys genetic variant. They performed a skin biopsy, which confirmed the accumulation of GOM in the basal layer of vascular smooth muscle cells, and neuroimaging tests showed diffuse white matter hyperintensities. In 2000, Ceroni et al. [61] described an Italian family with eight affected members who exhibited autosomal dominant migraine with prolonged visual, sensory, motor, and aphasic auras. These symptoms were associated with white matter abnormalities following brain MRI. All living affected members carry the *NOTCH3* (p.Arg153Cys) mutation previously reported with CADASIL. The same situation was described by Guey et al. [62] in 2016. The patient identified with the p.Arg153Cys variant suffered from MA, specifically an atypical aura that included a motor aura. In our study, a woman with the p.Arg153Cys reported headaches without aura or motor and visual abnormalities. Her headaches lasted for <24/h and occurred 1–2 times a month. However, similarly to what was described by Zhu et al. [63], she had ischemic changes in the periventricular white matter, which were made apparent upon MRI imaging. However, it is worth emphasizing that the benign variant p.Thr101= was also present in our patient.

The likely pathogenic variant p.Cys144Arg was detected in only one CADASIL patient, a 53-year-old female who had been reporting headaches for only a year (late-onset at 52). However, she experienced headaches frequently (>4 times a month). In addition, extensive vascular changes in the white matter were detected in the patient. No visual aura, motor abnormalities, or other symptoms related to MA or MO were described in this patient. However, research on this variant is very limited. As described in the ClinVar database, the p.Cys144Arg variant disrupts a cysteine residue in an EGF-like repeat domain, which is important for the structure of the protein. Therefore, it is expected to severely affect the protein’s function [51]. Due to the lack of literature data, we assume that this variant may be another genetic variant of probable importance in the diagnosis of CADASIL, but so far, no efforts have been devoted to understanding the influence of this variant in relation to headaches in a patient with CADASIL. Moreover, two benign variants of the *NOTCH3* gene were also identified in this patient: p.Ala202= and p.Thr101=.

A benign variant, p.Thr101=, of the *NOTCH3* gene has been previously studied in the context of CADASIL [64,65,66,67,68]. The results of these types of studies are varied. Keat Wei et al. [69] and González-Giraldo et al. [70] concluded that this mild variant probably does not play a major role in the pathogenesis of CADASIL. He concluded that due to the high prevalence of this variant in the general population, it does not affect the further development of CADASIL in patients suffering from headaches. According to the Ensemble.org database [71], the frequency of the wild-type T allele in the European population is 85%, and the frequency of the alternative C allele in the European population is 15%. The results of our study confirmed its frequent occurrence in the Polish population. We found its presence in 8 of the 30 people tested. In addition, we confirmed the correlation between headaches and CADASIL in approximately 25% of those tested who have been diagnosed with ischemic events. We showed that its presence may be associated with MO (13% of patients). In the Polish population, most subjects with this genetic variant had symptoms of MA (61% of patients). We also showed that the clinical picture pertaining to headaches is not homogenous and can include TTH and other headaches in addition to MA and MO.

The second benign variant, p.Ala202=, was described as probably not a risk factor for migraine in CADASIL because the polymorphic A allele was more common in healthy individuals than in those with migraine [31]. However, in our study, we identified this variant in three people of the same family who had different headache courses in CADASIL. The 46-year-old mother in this family has been suffering from MA for 10 years (onset 36), while her 18-year-old son has suffered from tension-type headaches for 10 years (onset at the age of 8). Both the mother and son have similar headaches that occur 2–3 times a month and last for several minutes. Menon et al. mentioned that CADASIL patients had episodes of headache among their symptoms [72]. Numerous bilateral periventricular ischemic changes were present only in the mother’s computed tomography results. The second, older son (21 years old) was shown to have the same genetic variant but has not had any headaches or other symptoms of CADASIL. Moreover, the mother’s illness began at the age of 36; thus, it could be associated with pregnancy and childbirth [24,25]. In turn, the younger son’s TTH appeared as early as 8 years of age due to the onset of puberty [73,74]. Both cases in the family appear to be hormonal.

The chr19:15192257 T>G variant was first reported by Coto et al. in 2006 [75]. The authors describe a 44-year-old patient with CADASIL symptoms, in whom this genetic variant was confirmed. Interestingly, the authors emphasize that the parents of this patient were neurologically healthy and did not carry this mutation. They also emphasize the need for further analyses of *NOTCH3* gene variants in people with CADASIL symptoms but with a family history. Despite reporting a new mutation in exon 4 of the *NOTCH3* gene, Coto et al. [75] did not provide a detailed clinical picture of the patient with this change. In our study, we described the patient’s CADASIL picture depending on the type of headache that was one of the symptoms.

A patient with a chr19:15192257 T>G genetic variant of the *NOTCH3* was diagnosed with MO. The onset of MO occurred at the age of 34. This patient’s MO attacks lasted several hours and appeared 1–2 times a month. The patient was reported to the neurological ward with symptoms of paresis of the upper limbs and increasing pain in the left eye socket, with an earlier diagnosis of strabismus. However, neuroimaging studies did not show ischemic changes. Moreover, numerous cases of migraines and strokes have been reported among family members of this patient.

It should be remembered that research on identifying genes involved in the pathogenesis of headaches is still ongoing [76,77,78]. However, understanding the genes directly involved in headaches may help to diagnose diseases in which headaches are an important symptom that often indicate the onset of an disease. Moreover, it should be remembered that various headaches may occur with CADASIL [79].

## 5. Conclusions

The present research study has shown that genetic variants of the *NOTCH3* gene may be associated with the presence or absence of headaches during CADASIL or several years before their onset as a harbinger of the disease. Moreover, if the same pathogenic or benign genetic change is present, even in the same family, headache symptoms may vary and present as MA and MO, TTH, and others. Studies with *NOTCH3* genetic variants as a diagnostic and therapeutic factor, especially in the context of headaches, should conduct further research on larger groups of people, including relatives and those without a family history of the condition.

## Figures and Tables

**Table 1:** Mutations in the *NOTCH3* gene.

Domain of NOTCH3 Protein	Mutations in the *NOTCH3* Gene
Signal peptide	L33 del, A34V
EGF-like 1	C43G, C43F, C49G, C49F, C49Y, R54C, S60C, C65S, C65Y, C67Y, W71C, R75W, R75P, C76R, C76W
EGF-like 2	77–83 del, 80–84 del, C87R, C87Y, R90C, C93F, C93Y, C93 dup, C106W, C108W, C108Y, C108R, R110C, R113Q, 114–120 del, C117F, S118C
EGF-like 3	C123F, C123Y, C128Y, C128G, C128F, G131C, R133C, C134W, D139V, R141C, F142C, C144F, C144S, C144Y, S145C, C146R, C146F, C146Y, G149C, Y150C, 153–155 del, R153C, C155S, C155Y
EGF-like 4, calcium-binding	C162S, C162W, R169C, H170R, G171C, C174F, C174R, C174Y, S180C, R182C, C183F, C183R, C183S, C185G, C185R, Y189C, C194F, C194R, C194S, C194Y
EGF-like 5	C201Y, C201R, A202E, C206Y, C206R, R207C, C209R, C212S, R213K, Y220C, C222G, C222Y, C224Y, C233S, C233Y, C233W
EGF-like 6, calcium-binding	239–253 del, V237M, C240S, C245R, C251R, C251S, C251G, Y258C, C260Y, C271F
EGF-like 7	S299C
EGF-like 8, calcium-binding	A319C, R332C, S335C, Y337C, C338R
EGF-like 9	C366W, CC379S, C379R, G382C, C388Y
EGF-like 10, calcium-binding	C395R, G420C, R421C, P426L, C428Y, C428S
EGF-like 11, calcium-binding	C435R, C440G, C440S, C446S, C446F, R449C, C455R, Y465C
EGF-like 12, calcium-binding	C484F, C484Y, C4884G, C495Y, P496L, S497L
EGF-like 13, calcium-binding	C511R, C516Y, G528C, R532C, C533Y, C542Y
Interdomain	R544C
EGF-like 14, calcium-binding	C549Y, C549R, H556R, R558C, C568Y, R578C, R578H
EGF-like 15, calcium-binding	A587C, C591R, C597W, R607C, R607H
EGF-like 16, calcium-binding	R640C, V644D
EGF-like 17, calcium-binding	G667C, R680H
EGF-like 18	Y710C, R728C
EGF-like 19	R767H, C775S
EGF-like 20 -> EGF-like 23, calcium-binding	-
EGF-like 24	G953C
EGF-like 25	C977S, S978R, F984C, R985C, C988Y, C997G
EGF-like 26	R1006C, C1015R, A1020P, Y1021C, W1028C, R1031C
EGF-like 27	G1058C, C1061Y, D1063C, R1076C
EGF-like 28	C1099Y, Y1106C, N1118Y
EGF-like 29, calcium-binding	H1133Q, C1157W
EGF-like 30, calcium-binding	V1183M
EGF-like 31	R1231C, H1235L, R1242H
EGF-like 32	C1250W, C1261R, C1261Y
EGF-like 33	Q1297L
EGF-like 34	P1357L
LNR 1 -> LNR 3	-
HD?	L1518M, L1547V, I1586V
RAM?	L1691E, G1710D, R1748H, V1762M, R1837H
ANK 1 -> ANK 5	A1850S, A1850D, V1952M, F1995C, P2033T, P2074L, R2109Q, A2223V

Source: https://www.uniprot.org/ (accessed on 10 August 2023), https://www.omim.org/entry/600276 (accessed on 10 August 2023)

**Table 2:** Demographic data of subjects with CADASIL.

Number of People (N = 30)		Female	N = 20	Male	N = 10
Age		43.6 ± 11.5		39.6 ± 15.8	
Age of CADASIL diagnosis		≤45 years old >45 years old	11 9	≤45 years old >45 years old	5 5
Headache		Yes	20	Yes	9
MA		Yes	10	Yes	4
MO		Yes	3	Yes	0
Other types of headaches (N = 12)	TTH (N = 2)	Yes	1	Yes	1
	Headache (N = 10)	Yes	6	Yes	4
Family history of headache		Yes	11	Yes	4
Genetic tests of *NOTCH3*		Yes	20	Yes	10
Stroke in family history		Yes	2	Yes	1
Neuroimaging changes (MRI/CT)		Vascular changes/ischemic stroke	8	Vascular changes/ischemic stroke	2

N—number of people, F—female, M—male, MA—migraine with aura, MO—migraine without aura, TTH—tension-type headache, MRI—magnetic resonance imaging, CT—computed tomography.

**Table 3:** Primer sequences for *NOTCH3* genetic variant analysis.

Genetic Variants of *NOTCH3* Gene	Primer Sequences	Exon	Temperature of Annealing	Product Size [Base Pair, bp]
**rs3815188** p.Thr101= **rs28937321** p.Trp71Cys	Forward: 5′GGCCTCAGARAGAGCTGAACC3′	3	65 °C	303 bp
	Reverse: 5′ACCCTCGATCTAAGGACCCC3′			
**rs1043994** p.Ala202=	Forward: 5′GATGGACGCTTCCTCTGCT3′	4	62 °C	300 bp
	Reverse: 5′CACCCCTCTGACTCTCCTGA3′			
**rs371491165** p.Gln151Glu **rs797045014** p.Arg153Cys **rs2893369** **rs28933697** p.Arg182Cys	Forward: 5′GATGGACGCTTCCTCTGCT3′	4	63 °C	203 bp
	Reverse: 5′CATGGTGAGGGTGCACAG3′			
**rs797045015** p.Asp105Gly **rs137852642** p.Arg133Cys **rs371491165** p.Gln151Glu	Forward: 5′AGTCTGGAGGGGAGGTAGTC3′	4	65 °C	217 bp
	Reverse: 5′CACCCGGCACTCATCCAC3′			
**rs864621965** p.Asp239_Asp253del	Forward: 5′GACCATCCTTGCCCCCTTC3′	5	65 °C	209 bp
	Reverse: 5′CACCTGGCGCATGTCCAC3′			
**rs35793356** p.Gly594=	Forward: 5′GGCCTCAGARAGAGCTGAACC3′	11 12	65 °C	600 bp
	Reverse: 5′ACCCTCGATCTAAGGACCCC3′			

**Table 4:** Genetic variants of the *NOTCH3* gene and the clinical characteristics of patients with CADASIL.

*NOTCH3* Genetic Variant	Clinical Significance	Number of Patients	Age of Patient [Mean Age ± SD or Single Results]	Type of Headache					Headache Attack Duration [Hours]			Number of Headache Attacks [Per Month]			Changes in the MRI/CT Image		References
				MA	MO	Other Types of Headaches		No Headache	<24	24–48	>48	1–2	3–4	>4	Vascular Changes/Ischemic Stroke	No Changes	
						TTH	Other										
p.Tyr189Cys	Pathogenic	3	34.0 ± 1.0	3	0	0	0	0	0	0	3	2	1	0	2	1	[49]
p.Arg153Cys	Pathogenic	1	63	0	0	0	1	0	1	0	0	1	0	0	1	0	[50]
p.Thr101=	Benign																
p.Cys144Arg	Likely pathogenic	1	53	0	0	0	1	0	1	0	0	0	0	1	1	0	[51]
p.Ala202= p.Thr101=	Benign																
p.Ala202=	Benign	16	39.9 ± 13.0	9	1	1	4	1	14	1	1	10	2	4	3	13	[52]
p.Thr101= and p.Ala202=	Benign	8	45.9 ± 13.5	5	1	1	1	0	4	3	1	3	1	4	2	6	[53]
chr19:15192257 T>G, exon 4, codon: 128, Cysteine	Not confirmed in the literature	1	45	0	1	0	0	0	1	0	0	1	0	0	0	1	Varsome page [48]

SD—standard deviation, MA—migraine with aura, MO—migraine without aura, TTH—tension-type headache, MRI—magnetic resonance imaging, CT—computed tomography. Fisher’s exact test was used, statistically significant differences between MA and other headaches (*p* < 0.05).

## Data Availability

Data is contained within the article.
