# Correction: Szymanowicz et al. Headache and *NOTCH3* Gene Variants in Patients with CADASIL. *Neurol. Int.* 2023, *15*, 1238–1252

**DOI:** 10.3390/neurolint16030038

**Published:** 2024-05-06

**Authors:** Oliwia Szymanowicz, Izabela Korczowska-Łącka, Bartosz Słowikowski, Małgorzata Wiszniewska, Ada Piotrowska, Ulyana Goutor, Paweł P. Jagodziński, Wojciech Kozubski, Jolanta Dorszewska

**Affiliations:** 1 Laboratory of Neurobiology, Department of Neurology, Poznan University of Medical Sciences, 61-701 Poznan, Polandikorcz@post.pl (I.K.-Ł.);; 2 Department of Biochemistry and Molecular Biology, Poznan University of Medical Sciences, 61-701 Poznan, Poland; 3 Faculty of Health Care, Stanislaw Staszic University of Applied Sciences in Pila, 64-920 Pila, Poland; 4 Department of Neurology, Specialistic Hospital in Pila, 64-920 Pila, Poland; 5 Chair and Department of Neurology, Poznan University of Medical Sciences, 61-701 Poznan, Poland

**Figure Description**

In the original publication [1], there was a mistake in the legend for Figure 1. Heterozygous variant in the *NOTCH3* gene, chr19:15192257 T>G.

The correct description appears below.

Correct description of Figure 1:

**Figure 1:**
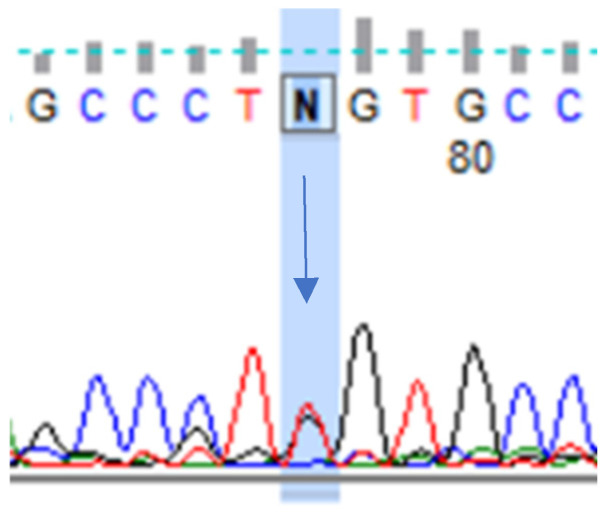
Heterozygous variant in the *NOTCH3* gene, chr19:15192257 T>G. A clinical characteristics of this variant not yet been described in the literature (analyzed according to the following database: https://varsome.com/) (accessed on 13 December 2023).

The authors corrected the name of the *NOTCH3* genetic variant (chr19:15192257 T>G). The authors also updated the Varsome website (to 13 December 2023).

The authors state that the scientific conclusions are unaffected. This correction was approved by the Academic Editor. The original publication has also been updated.

**Error in Table**

In the original publication, there was a mistake in Table 4. Genetic variants of the *NOTCH3* gene and the clinical characteristics of patients with CADASIL as published.

Table 4 included an error regarding a *NOTCH3* genetic variant name (chr19:15192258 G>T) and its localization (exon 4, codon: 127) and coded protein (proline). The clinical significance described as not described in the literature was also incorrect.

The corrected Table 4. Genetic variants of the *NOTCH3* gene and the clinical characteristics of patients with CADASIL appears below.

**Table 4:** Genetic variants of the *NOTCH3* gene and the clinical characteristics of patients with CADASIL.

*NOTCH3* Genetic Variant	Clinical Significance	Number of Patients	Age of Patient [Mean Age ± SD or Single Results]	Type of Headache					Headache Attack Duration [Hours]			Number of Headache Attacks [Per Month]			Changes in the MRI/CT Image		References
				MA	MO	Other Types of Headaches		No Headache	<24	24–48	>48	1–2	3–4	>4	Vascular Changes/Ischemic Stroke	No Changes	
						TTH	Other										
p.Tyr189Cys	Pathogenic	3	34.0 ± 1.0	3	0	0	0	0	0	0	3	2	1	0	2	1	[49]
p.Arg153Cys	Pathogenic	1	63	0	0	0	1	0	1	0	0	1	0	0	1	0	[50]
p.Thr101=	Benign																
p.Cys144Arg	Likely pathogenic	1	53	0	0	0	1	0	1	0	0	0	0	1	1	0	[51]
p.Ala202= p.Thr101=	Benign																
p.Ala202=	Benign	16	39.9 ± 13.0	9	1	1	4	1	14	1	1	10	2	4	3	13	[52]
p.Thr101= and p.Ala202=	Benign	8	45.9 ± 13.5	5	1	1	1	0	4	3	1	3	1	4	2	6	[53]
chr19:15192257 T>G, exon 4, codon: 128, Cysteine	Not confirmed in the literature	1	45	0	1	0	0	0	1	0	0	1	0	0	0	1	Varsome page [48]

SD—standard deviation, MA—migraine with aura, MO—migraine without aura, TTH—tension-type headache, MRI—magnetic resonance imaging, CT—computed tomography. Fisher’s exact test was used, statistically significant differences between MA and other headaches (*p* < 0.05).

The authors corrected the *NOTCH3* genetic variant name, localization, and protein (chr19:15192257 T>G, exon 4, codon: 128, cysteine). The authors also corrected the clinical significance of this genetic variant—the clinical significance has not been confirmed in the literature so far. The characterization of the patient’s headache remained the same.

The authors state that the scientific conclusions are unaffected. This correction was approved by the Academic Editor. The original publication has also been updated.

**Missing Citation**

In the original publication [1], Coto, E.; Menéndez, M.; Navarro, R.; García-Castro, M.; Alvarez, V. A new de novo Notch3 mutation causing CADASIL. *European Journal of Neurology* 2006, *13*, 628–631 was not cited.

The citation has now been inserted in the Discussion section and References section and should read as follows:

The chr19:15192257 T>G variant was first reported by Coto et al. in 2006 [75]. The authors describe a 44-year-old patient with CADASIL symptoms, in whom this genetic variant was confirmed. Interestingly, the authors emphasize that the parents of this patient were neurologically healthy and did not carry this mutation. They also emphasize the need for further analyses of *NOTCH3* gene variants in people with CADASIL symptoms but with a family history. Despite reporting a new mutation in exon 4 of the *NOTCH3* gene, Coto et al. [75] did not provide a detailed clinical picture of the patient with this change. In our study, we described the patient’s CADASIL picture depending on the type of headache that was one of the symptoms.

The authors added information about chr19:15192257 T>G from the first publication by Coto et al. in 2006.

The authors state that the scientific conclusions are unaffected. This correction was approved by the Academic Editor. The original publication has also been updated.

**Text Correction**

There were errors in the text of the original publication. In the text of the Abstract, Results, and Discussion sections, there were mistakes in the genetic variant’s name, its localization, and coded protein. Because of the sequencing error, there were also mistakes in the Varsome interpretation.

A correction has been made to the following:

1. Abstract section

We described three variants as pathogenic/likely pathogenic (p.Tyr189Cys, p.Arg153Cys, p.Cys144Arg) and two benign variants (p.Ala202=, p.Thr101=) in the *NOTCH3* gene and also presented the *NOTCH3* gene variant (chr19:15192257 T>G). Clinical features including headache associated with *NOTCH3* (chr19:15192257 T>G) are described for the first time.

The authors corrected the *NOTCH3* gene variant name and added information about its data in the literature—this variant was only reported in one publication, but the authors added a specific clinical characterization of this variant.

2. Results section

Moreover, we identified the *NOTCH3* gene mutation (chr19:15192257/c.382T>G p.Cys128Gly), displayed in Figure 1, which has only been mentioned once as a “de novo mutation” in CADASIL patients in the literature to date, but this is the first time we have described the clinical features including headache associated with this variant.

According to the Varsome database (accessed on 13 December 2023), the variant chr19:15192257 T>G of the *NOTCH3* gene, identified in exon 4, is located in codon 128 and encodes a cysteine [48].

The chr19:15192257 T>G variant was found in one patient (female, 45 years old) who reported episodes of MO from the age of 34 (>10 years of MO).

The authors corrected data about the chr19:15192257 variant. The authors corrected its localization and change in the protein. The authors also added the information that this variant was not described in a clinical way.

3. Discussion

Three pathogenic/likely pathogenic variants, two benign variants, and one variant of the *NOTCH3* gene (chr19:15192257 T>G) have been reported in the literature [48], but the detailed clinical significance last variant and clinical picture of a patient with this variant has not been presented.

The authors corrected the genetic variant name.

The chr19:15192257 T>G variant was first reported by Coto et al. in 2006 [75]. The authors describe a 44-year-old patient with CADASIL symptoms, in whom this genetic variant was confirmed. Interestingly, the authors emphasize that the parents of this patient were neurologically healthy and did not carry this mutation. They also emphasize the need for further analyses of *NOTCH3* gene variants in people with CADASIL symptoms but with a family history. Despite reporting a new mutation in exon 4 of the *NOTCH3* gene, Coto et al. [75] did not provide a detailed clinical picture of the patient with this change. In our study, we described the patient’s CADASIL picture depending on the type of headache that was one of the symptoms.

A patient with a chr19:15192257 T>G genetic variant of the *NOTCH3* was diagnosed with MO.

The authors added information about chr19:15192257 T>G from the first publication by Coto et al. in 2006.

The authors state that the scientific conclusions are unaffected. This correction was approved by the Academic Editor. The original publication has also been updated.

**References**

The authors corrected references. The authors updated the Varsome database website to 13 December 2023 and updated the website’s link.

48. Varsome.com. Available online: https://varsome.com/position/hg38/19:15192257 (accessed on 13 December 2023).

The authors also added the 75th publication as a reference where the chr19:15192257 T>G variant was mentioned first:

75. Coto, E.; Menéndez, M.; Navarro, R.; García-Castro, M.; Alvarez, V. A new de novo Notch3 mutation causing CADASIL. *Eur. J. Neurol.* **2006**, *13*, 628–631.

With this correction, the order of some references has been adjusted accordingly. The authors state that the scientific conclusions are unaffected. This correction was approved by the Academic Editor. The original publication has also been updated.
